# Supplementary material for: Plant cysteine oxidases are dioxygenases that directly enable arginyl transferase-catalysed arginylation of N-end rule targets
Source: Nat Commun. 2017 Mar 23;8:14690. doi: 10.1038/ncomms14690 (PMC5376641; doi:10.1038/ncomms14690)
Supplement: Supplementary Information — Supplementary Figures, Supplementary Tables and Supplementary References [file ncomms14690-s1.pdf]

Supplementary Figures

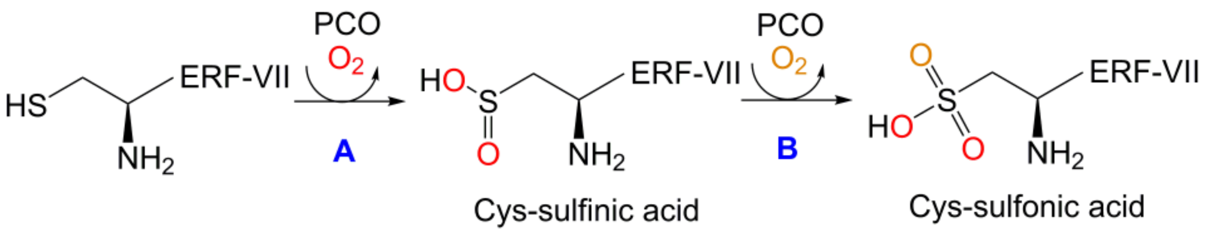

Supplementary Figure 1 | Scheme representing potential L-Cys oxidation reactions according to principles of the Arg/Cys branch of the N-end rule pathway in animals and plants.[1-3] N-terminal L-Cys residues must be oxidised to L-Cys sulfinic (**A**) or L-Cys sulfonic acid (**B**) prior to recognition by arginyl transferases. We report that the PCOs only catalyze reaction **A**, oxidation to Cys-sulfinic acid.

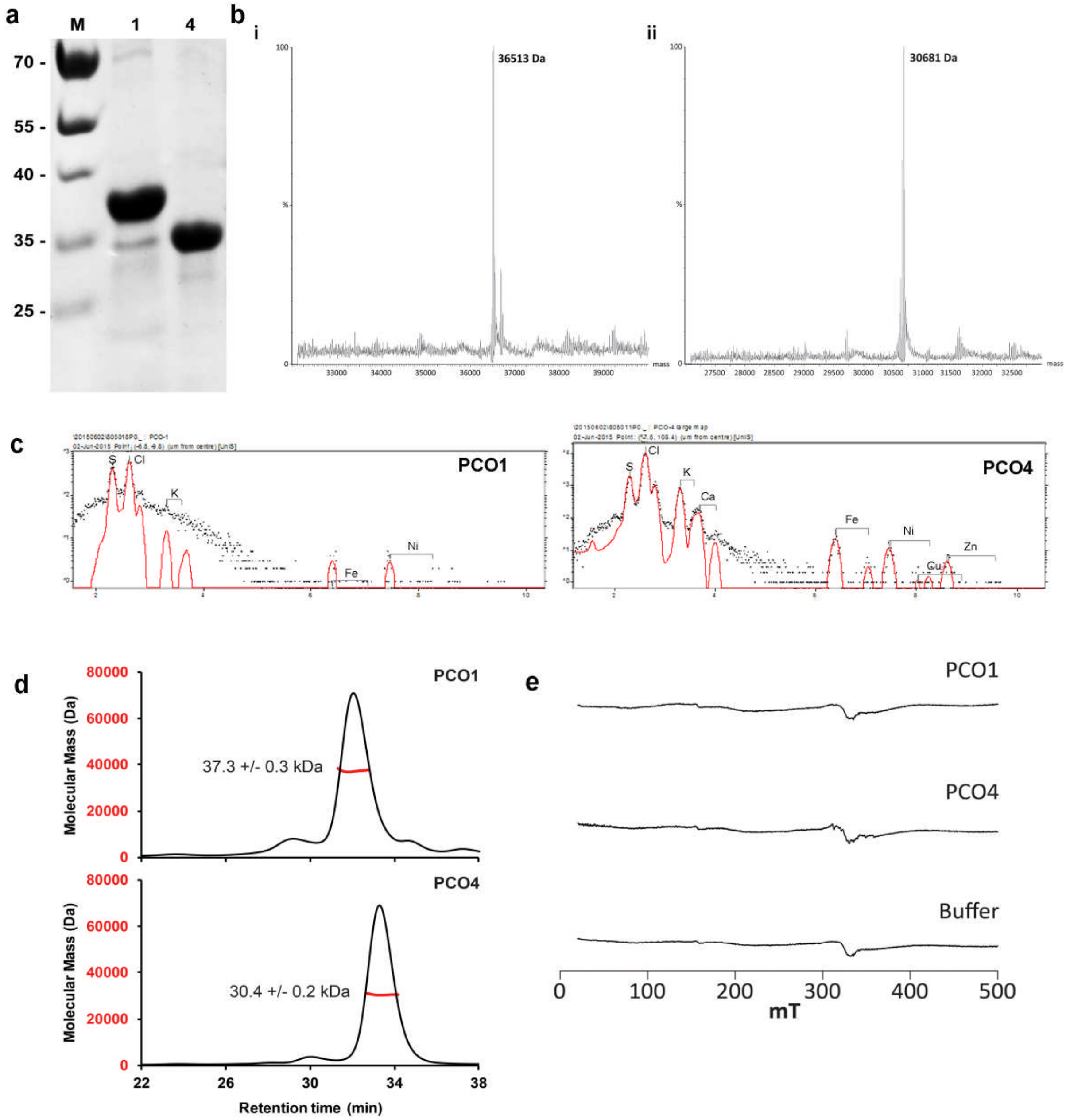

Supplementary Figure 2 | Purification and characterisation of recombinant *Arabidopsis* PCO1 and PCO4 **(a)** SDS-PAGE gel showing 6xHis:PCO1 (1) and 6xHis:PCO4 (4) with purity judged to be ~90%. M = molecular weight marker, kDa; **(b)** Liquid Chromatography-Mass Spectra (LC-MS) confirming the identity of recombinantly produced (i) PCO1 and (ii) PCO4. PCO1 predicted mass 36510 Da, observed mass 36513 Da; PCO4 predicted mass 30680 Da, observed mass 30681 Da; (**c**) Analysis of metal content (relative to the number of sulphur atoms) in PCO1 and PCO4 was determined by MicroPIXE (particle-induced X-ray emission with a micro-focussed beam[4]), and revealed 0.3 (± 0.12) iron atoms/molecule for PCO1 and 0.31 (± 0.015) iron atoms/molecule for PCO4 (n=3 and n=4, respectively). Both

PCO1 and PCO4 also had a high nickel content (0.45 ± 0.05 and 0.22 ± 0.02 atoms/molecule, respectively), presumably due to the Ni^{2+}-affinity purification procedure. No other metals were present in PCO1, while PCO4 contained a small level of zinc (0.13 ± 0.02 atoms/molecule) and copper at the limit of detection (0.033± 0.006 atoms/molecule). The buffer was clean of any metals. Representative PIXE spectra (X-ray energy against log counts) are shown for PCO1 and PCO4, which had been buffer exchanged from NaCl/Tris HCl into ammonium acetate to avoid overlap of the sulphur and chlorine peaks. Spectra were recorded using a 2 µm diameter beam of 2.5 MeV protons. (**d**) Multiple angle light scatter (MALS) experiments were performed during size exclusion chromatography on an analytical Superdex 200 10/300 GL column (GE Healthcare) equilibrated with 50 mM Tris pH 7.5, 400 mM NaCl. Elution was monitored via online static light-scattering (DAWN HELEOS 8+, Wyatt Technology), differential refractive index (Optilab rEX, Wyatt Technology) and UV (SPD-20A, Shimadzu) detectors. Data were analysed using the ASTRA software package (Wyatt Technology); (**e**) Continuous wave EPR spectra of PCO1 (100 µM), PCO4 (66 µM) and buffer only (250 mM NaCl, 50 mM Tris HCl pH 7.5) do not reveal significant Fe(III) content in either PCO1 or PCO4, indicative that enzyme-associated metal is in the Fe(II) form. Spectra were measured at 13K and a frequency of 9.39 GHz with a modulation amplitude of 0.4 mT and a microwave power of 2 mW in a Bruker EMX spectrometer.

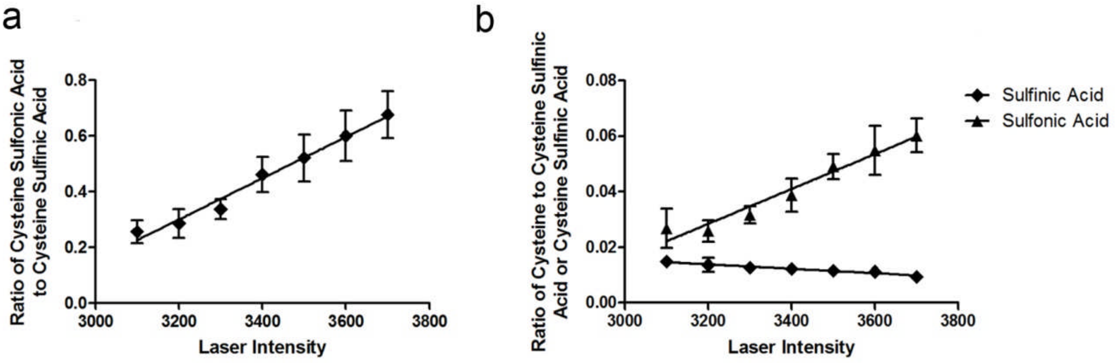

Supplementary Figure 3 | The effect of laser intensity on Matrix Assisted Laser Desorption/Ionisation-Mass Spectrometry (MALDI-MS) detection of (a) products of the PCO1/4-catalyzed RAP2$_{2\text{-}11}$ oxidation and (b) RAP2$_{2\text{-}11}$ only. (**a**) On analysis of products of the PCO-catalyzed reaction, the ratio of +48 Da:+32 Da peptidic products increased with laser intensity, indicating that conversion of the +32 Da to the +48 Da product (presumably Cys-sulfinic acid oxidation to Cys-sulfonic acid) is a laser-induced process. (**b**) Laser-induced conversion of unmodified RAP2$_{2\text{-}11}$ peptide to products with mass increases of +32 Da and +48 Da was much less significant, although a weak correlation (relative to that seen in panel A) was observed between laser intensity and Cys-sulfonic acid formation.

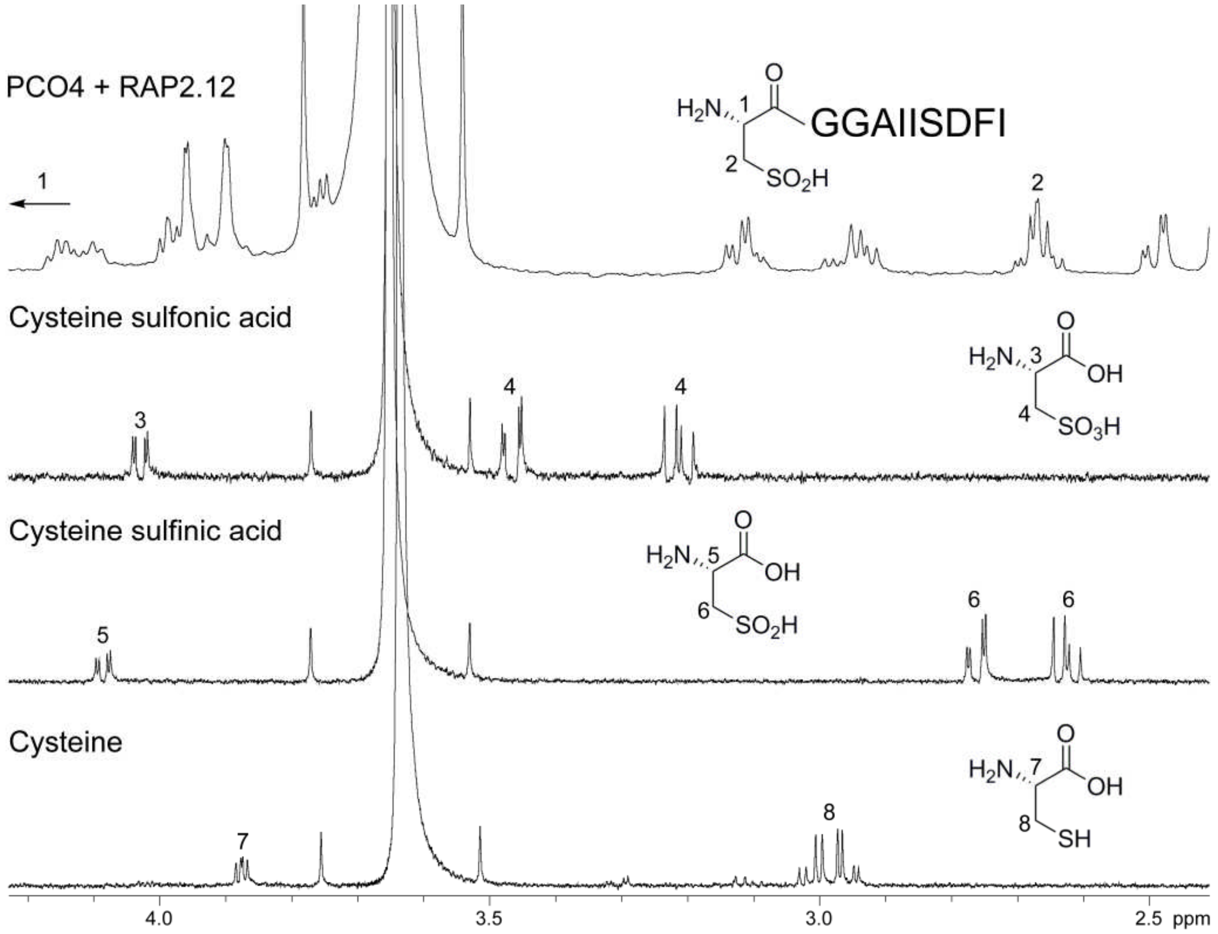

Supplementary Figure 4 | ^{1}H NMR spectra of cysteine, cysteine sulfonic acid, cysteine sulfonic acid and a reaction mixture containing RAP2$_{2\text{-}11}$ and PCO4. ^{1}H NMR spectra of L-Cys, L-Cys sulfinic acid and L-Cys sulfonic acid standards were generated by preparing each standard to 1 mM (in the presence of 10% D_2O) in a microcentrifuge tube (75 µL total volume) followed by transfer to 2mm diameter NMR tubes and analysis using a 600 MHz NMR spectrometer. Comparison of the proton resonances corresponding to b-cysteinyl protons of the PCO-catalyzed RAP2$_{2\text{-}11}$ modification (see main text **Figure 3b**) with those of the oxidized Cys amino acids support the enzymatic conversion of the RAP2$_{2\text{-}11}$ *N*-terminal L-Cysteine to L-Cysteine sulfinic acid. ^{1}H-resonances assigned to the β-cysteinyl protons are as follows: L-Cysteine at 2.96 ppm (dd, 1H, J_1 = 4.0 Hz, J_2 = 14.5 Hz), 3.01 ppm (dd, 1H, J_1 = 6.0 Hz, J_2 = 14.5 Hz), L-Cysteine sulfinic acid at 2.62 ppm (dd, 1H, J_1 = 10.0 Hz, J_2 = 14.0 Hz), 2.76 ppm (dd, 1H, J_1 = 3.0 Hz, J_2 = 14.0 Hz) and L-Cysteine sulfonic acid at 3.21 ppm (dd, 1H, J_1 = 11.0 Hz, J_2 = 15.0 Hz), 3.47 ppm (dd, 1H, J_1 = 2.5 Hz, J_2 = 15.0 Hz). J_1 represents the coupling constant to the alpha cysteinyl protons, whereas J_2 represents the coupling between the two beta protons.

a

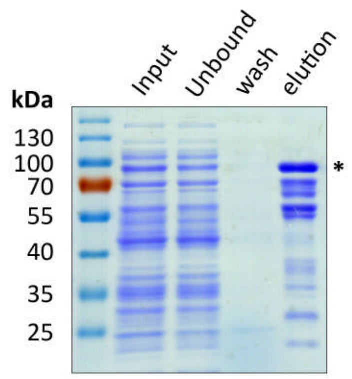

b

MSLKNDASSS HDGGSNRESV IDDHGRRKST CGYCKSPARS SISHGLSAQT LTVYDYQALI

DRGWRRSGTY LYKHEMDKTC CPPYTIRLKA SDFVPTKEQQ RVSRRLERFL DGKLDVQPRE

O C O
QRGASSSGDV SDTRRKTLGA AKSEENKKVE AVMDDLSKNI DQAVQLCIRS GEFPSNMQIP

KASVKKVFCA RRKKLAEGTE QILYTSNIAF PIAAAIKRIQ TSEKEGINSA EGNRLSPETI

O O
SEMLLSAMHK VGETPDVSIK VCKGHINFLS SAKDSFSDRD VVPNGNISRG ANSLDGSETL

HAKKDSENHQ ARKRKLEIHL KRSSFDPEEH ELYKRYQLKV HNDKPGHVVE SSYRRFLVDS

C C
PLIDVQPSGD EKVPPCGFGS FHQQYRIDGR LIAVGVVDIL PKCLSSVYLF WDPDYAFLSL

C C C
GKYSAIQEIN WVIENQARCP SLQYYYLGYY IHSCSKMRYK AAYRPSELLC PLRFQWVPFE

VARPMLDKKP YVILSDIAIS HNQCSLLAGA SETLVEPAAS EHEDMEQGET NDNFMGCSDE

DEDEDEDDDD DDDDDEEMYE TESEDSHIES DPGSKDNDIN NILIGLYGSQ YRYKEMRQII

O
TPVGRKQLEP MLQSYRKVVG AELSERMVYE IN

Supplementary Figure 5 | Purification and sequence verification of recombinant *Arabidopsis* ATE1. (a) SDS-PAGE gel of recombinant *Arabidopsis* 6xHis:ATE1 purification fractions (for expression and purification conditions, see Materials and Methods). 6xHis:ATE1 is expected at 74 kDa but runs slower. The protein band corresponding to experimentally active protein is seen at approximately 90 kDa (asterisk). **(b)** Sequence

coverage of recombinant ATE1 (90 kDa protein band) from peptides identified in an LC-MS/MS analysis following in-gel trypsin digestion of the band marked with an asterisk. A 30 minute LC gradient employing C18 reverse phase chemistry and a DDA scan strategy wherein 20 MS/MS spectra were acquired per MS full scan was used on an Orbitrap Velos Pro mass spectrometer. Peptides were identified by database search using the Mascot software linked to Proteome Discoverer. The false discovery rate was estimated using the common target-decoy database search strategy. Sequences highlighted in green represent peptides identified with an FDR cut-off of <1%. The sequence highlighted in red was identified as a statistically not significant peptide spectral match (PSM). Overall coverage with 99% confidence is 64% of the total sequence and verified the ATE1 production (**Supplementary Table 3**). C: Carbamidomethyl; O: Oxidation.

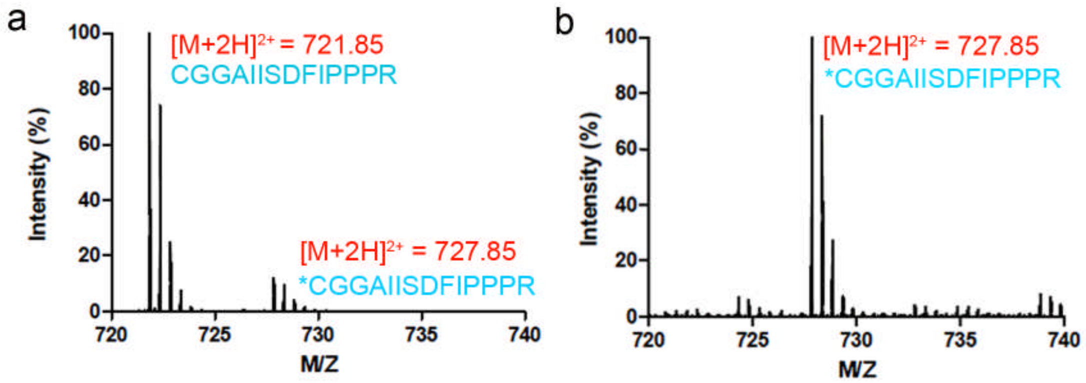

Supplementary Figure 6 | LC-MS spectra of RAP2$_{2\text{-}15}$ incubated in the presence of 50 mM HEPES and 1 mM DTT (no enzyme) for (a) 1 hour and (b) overnight. A RAP2$_{2\text{-}15}$ peptide was incubated in the presence of 50 mM HEPES and 1 mM DTT to demonstrate that these buffer conditions induce a +12 Da mass shift upon prolonged incubation (as required for the arginylation assay) in the absence of PCO-catalyzed oxidation (note peptide is observed in the doubly charged mass state). This suggests modification to the N-terminal cysteine, which could be a result of formaldehyde-induced thiazolidine formation[5].

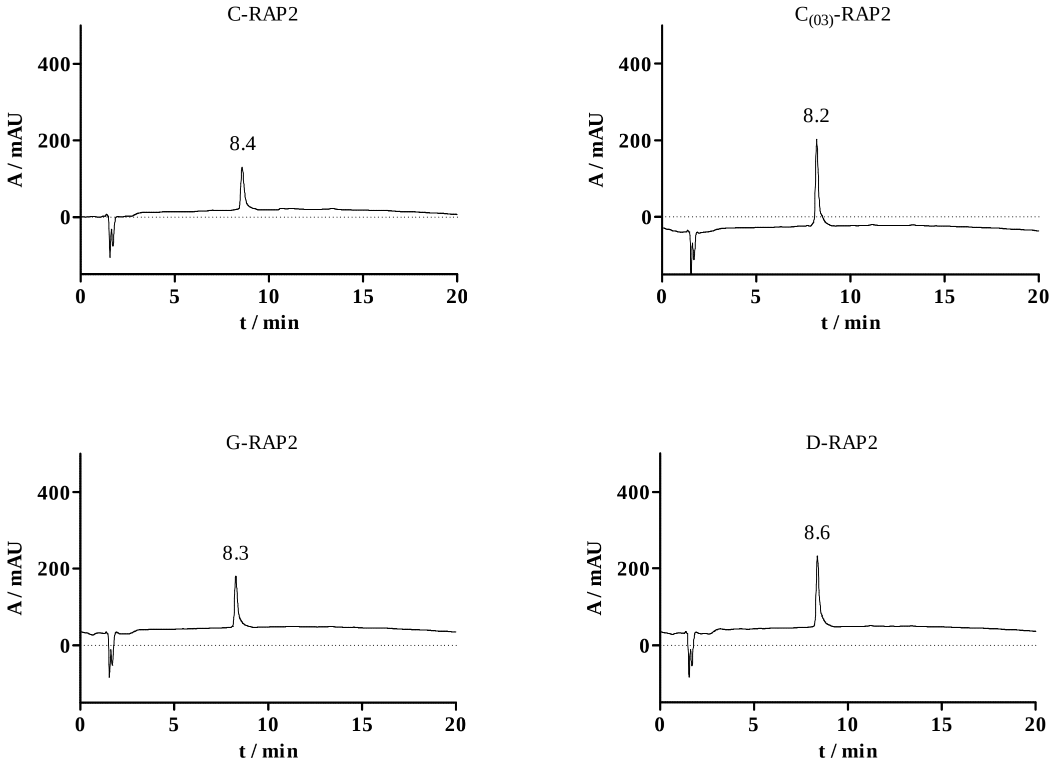

Supplementary Figure 7 | | High Performance Liquid Chromatography (HPLC) chromatograms showing purity of C/C$_{(O3)}$/D/G-RAP2$_{2\text{-}13}$ peptides synthesized for arginylation assays. Spectra of peptides were collected at 210 nm with a gradient of 20-60 % ACN in 20 min.

Supplementary Tables

Supplementary Table 1 | *Arabidopsis thaliana* ERF-VII N-termini.

Arabidopsis ERF-VII	N-terminal sequence
HRE1	MCGGAVISDYIAPEKIARSS…
HRE2	M**CGGAIISDFI**WSKSESEPS…
RAP2.12	M**CGGAIISDFI**PPPRSRRVT…
RAP2.2	M**CGGAIISDFI**PPPRSLRVT…
RAP2.3	MCGGAIISDYAPLVTKAKGR…

The 10-mer peptide substrate predominantly used in this study (underlined and bold) corresponds to $HRE2_{2\text{-}11}$, $RAP2.12_{2\text{-}11}$ and $RAP2.2_{2\text{-}11}$, and is collectively termed $RAP_{2\text{-}11}$. *In planta*, N-terminal Met residues are co-translationally cleaved by methionine amino-peptidases[6,7], exposing N-terminal Cys residues. The 12-mer sequence used in the coupled oxidation-arginylation assay is derived from RAP2.2. and RAP2.12 (X-GGAIISDFIPP(PEG)K(biotin)).

Supplementary Table 2 | Peptide sequences and characteristics used for arginylation reactions.

X	molecular formula	mw [g*mol^{-1}]	calculated m/z*	found m/z**	yield***
**C**	$C_{76}H_{122}N_{18}O_{21}S_2$	1688.02	845.01 / 563.67	1687.8 / 844.6 / 563.7	55 %
**$C_{(O3)}$**	$C_{76}H_{122}N_{18}O_{24}S_2$	1736.03	869.02 / 579.68	1736.1 / 868.6 / 579.8	44 %
**G**	$C_{75}H_{120}N_{18}O_{21}S1$	1641.93	824.97 / 548.31	1641.8 / 821.6 / 548.3	49 %
**D**	$C_{77}H_{122}N_{18}O_{23}S1$	1699.96	850.98 / 567.65	1699.8 / 850.6 / 567.4	69 %

X = N-terminal amino, followed by GGAIISDFIPP(PEG)K(biotin)-NH_2, amino acids in one letter code, PEG: polyethylene glycol (8-amino-3,6-dioxaoctanoyl)

*calculated m/z ratio for multi charged ions ($[M+nH]^{n+}$)

** experimentally determined m/z ratio for multi charged ions ($[M+nH]^{n+}$)

***yield after preparative HPLC purification

Supplementary Table 3 | Identified peptides of recombinant ATE1 from MS/MS.

Sequence	# PSMs	Protein Group Accessions	Modifications	ΔCn	IonScore	Exp Value	Charge	MH+ [Da]	ΔM [ppm]	RT [min]	# Missed Cleavages
FLVDSPLIDVQPSGDEK	18	AT5G05700.1		0,0000	105	2,87645E-10	2	1858,94097	-1,30	28,99	0
DNDINNILIGLYGSQYR	26	AT5G05700.1		0,0000	96	2,99416E-09	2	1967,97856	-1,84	30,99	0
YSAIQEINWVIENQAR	19	AT5G05700.1		0,0000	91	8,40202E-09	2	1933,97368	-1,57	35,92	0
LAEGTEQILYTSNIAFPIAAAIK	19	AT5G05700.1		0,0000	91	3,52751E-09	2	2434,32182	-0,45	30,84	0
SSISHGLSAQTLTVYDYQALIDR	11	AT5G05700.1		0,0000	89	1,09518E-08	2	2538,28081	-1,07	31,85	0
KVEAVMDDLSK	5	AT5G05700.1		0,0000	89	1,84476E-08	2	1234,63481	-0,02	30,30	1
IQTSEKEGINSAEGNR	26	AT5G05700.1		0,0000	88	1,43361E-08	2	1732,84319	-1,69	28,02	1
GASSSGDVSDTR	34	AT5G05700.1		0,0000	87	5,3894E-09	2	1138,49639	-0,66	16,50	0
VPPcGFGSFHQQYR	10	AT5G05700.1	C4(Carbamidomethyl)	0,0000	86	1,70349E-08	2	1679,78349	5,19	30,12	0
cPSLQYYYLGYYIHScSK	13	AT5G05700.1	C1(Carbamidomethyl); C16(Carbamidomethyl)	0,0000	85	1,30993E-08	2	2302,03838	3,31	31,01	0
LIAVGVVDILPK	14	AT5G05700.1		0,0000	81	1,10687E-08	2	1236,79289	0,19	31,24	0
GANSLDGSETLHAK	31	AT5G05700.1		0,0000	81	6,59863E-08	2	1399,68010	-0,83	34,76	0
LSPETISEMLLSAMHK	3	AT5G05700.1		0,0000	80	1,12647E-07	2	1786,90495	-1,63	33,94	0
NIDQAVQLcIR	22	AT5G05700.1	C9(Carbamidomethyl)	0,0000	80	1,09703E-07	2	1329,69353	-0,66	37,86	0
LAEGTEQILYTSNIAFPIAAAIKR	1	AT5G05700.1		0,0000	80	3,19374E-08	3	2590,42563	0,62	31,95	1
GASSSGDVSDTRRK	1	AT5G05700.1		0,0000	78	1,44756E-07	2	1422,69487	1,15	17,46	2
SSFDPEEHELYKR	6	AT5G05700.1		0,0000	77	1,25289E-07	2	1636,75603	-2,57	30,68	1
KVEAVmDDLSK	11	AT5G05700.1	M6(Oxidation)	0,0000	74	5,2309E-07	2	1250,62541	-3,47	29,67	1
SEENKKVEAVMDDLSK	7	AT5G05700.1		0,0000	71	9,64242E-07	2	1821,89018	0,12	31,12	2
KVVGAELSER	10	AT5G05700.1		0,0000	71	1,02307E-06	2	1087,61089	0,21	32,43	1
RFLVDSPLIDVQPSGDEK	3	AT5G05700.1		0,0000	70	9,78867E-07	2	2015,04057	-1,95	30,60	1

EGINSAEGNR	14	AT5G05700.1		0,0000	67	6,85596E-07	2	1046,48613	-0,05	15,76	0
SGEFPSNMQIPK	25	AT5G05700.1		0,0000	66	2,09322E-06	2	1334,64189	0,68	23,86	0
VVGAELSER	27	AT5G05700.1		0,0000	66	3,37995E-06	2	959,51659	0,93	20,39	0
SSFDPEEHELYK	20	AT5G05700.1		0,0000	64	1,17031E-06	2	1480,65837	-0,50	35,35	0
VEAVMDDLSK	19	AT5G05700.1		0,0000	64	3,1453E-06	2	1106,54021	0,30	25,60	0
SGEFPSNmQIPK	12	AT5G05700.1	M8(Oxidation)	0,0000	63	4,37488E-06	2	1350,63457	-0,98	20,76	0
LSPETISEmLLSAmHK	2	AT5G05700.1	M9(Oxidation); M14(Oxidation)	0,0000	63	6,01554E-06	2	1818,89714	-0,30	30,42	0
NDASSSHDGGSNR	17	AT5G05700.1		0,0000	62	9,33465E-07	2	1303,52458	-0,94	18,38	0
ASDFVPTK	22	AT5G05700.1		0,0000	61	8,04028E-06	2	864,44664	0,51	21,04	0
VEAVmDDLSK	5	AT5G05700.1	M5(Oxidation)	0,0000	59	6,80537E-06	2	1122,53276	-1,80	18,92	0
SGTYLYK	25	AT5G05700.1		0,0000	59	6,62222E-06	2	831,42717	2,97	23,41	0
VGETPDVSIK	29	AT5G05700.1		0,0000	59	1,24115E-05	2	1044,55766	0,42	26,07	0
QLEPmLQSYR	7	AT5G05700.1	M5(Oxidation)	0,0000	58	1,24442E-05	2	1280,62651	-3,03	22,87	0
ESVIDDHGR	13	AT5G05700.1		0,0000	56	9,32866E-06	2	1027,48039	0,03	34,62	0
KLAEGTEQILYTSNIAFPIAAAIK	7	AT5G05700.1		0,0000	54	1,37814E-05	3	2562,42020	0,90	35,39	1
QLEPMLQSYR	11	AT5G05700.1		0,0000	54	5,31508E-05	2	1264,63542	-0,05	26,97	0
cLSSVYLFWDPDYAFLSLGK	2	AT5G05700.1	C1(Carbamidomethyl)	0,0000	53	3,93519E-05	3	2381,15073	-0,67	35,16	0
VATVSLPR	28	TRYP_PIG		0,0000	53	3,26092E-05	2	842,50963	0,19	39,29	0
FLVDSPLIDVQPSGDEKVPPcGFGSFHQQYR	3	AT5G05700.1	C21(Carbamidomethyl)	0,0000	52	3,84939E-05	4	3519,69155	-2,49	32,11	1
TccPPYTIR	20	AT5G05700.1	C2(Carbamidomethyl); C3(Carbamidomethyl)	0,0000	49	6,109E-05	2	1167,52519	-2,88	24,19	0
SEENKKVEAVmDDLSK	4	AT5G05700.1	M11(Oxidation)	0,0000	47	0,000166207	3	1837,88248	-1,31	30,49	2
KDSENHQAR	1	AT5G05700.1		0,0000	46	0,000170336	2	1084,51286	-0,17	4,90	1
QIITPVGR	19	AT5G05700.1		0,0000	46	6,88406E-05	2	883,53691	1,01	24,42	0
LSPETISEMLLSAmHK	2	AT5G05700.1	M14(Oxidation)	0,0000	43	0,00066891	2	1802,90495	1,21	31,84	0
KSTcGYcK	12	AT5G05700.1	C4(Carbamidomethyl);	0,0000	40	0,000130948	2	1003,43346	-0,13	6,51	1

			C7(Carbamidomethyl)								
IEISELNR	3	P35908;P04264		0,0000	38	0,001585318	2	973,53160	0,28	24,47	0
AAYRPSELLcPLR	9	AT5G05700.1	C10(Carbamidomethyl)	0,0000	38	0,002363095	3	1545,82120	0,34	31,07	0
GHINFLSSAK	9	AT5G05700.1		0,0000	36	0,002282634	2	1073,57366	-0,19	32,95	0
STcGYcK	13	AT5G05700.1	C3(Carbamidomethyl); C6(Carbamidomethyl)	0,0000	36	0,000381152	2	875,33879	0,20	11,74	0
KQLEPmLQSYR	3	AT5G05700.1	M6(Oxidation)	0,0000	35	0,003708669	2	1408,71807	-5,18	29,44	1
GASSSGDVSDTRR	2	AT5G05700.1		0,0000	30	0,005838388	3	1294,59995	1,30	25,18	1
RIQTSEK	1	AT5G05700.1		0,0000	28	0,017656509	2	861,47899	0,12	5,91	1
EATISIQSAIRK	2	AT4G21820.1		0,0000	26	0,01596452	2	1316,75676	2,63	29,76	1
REYKVVMmmDFR	1	AT3G23420.1	M8(Oxidation); M9(Oxidation)	0,0000	25	0,020366357	3	1636,75870	-3,55	30,56	2
HEMDKTccPPYTIR	2	AT5G05700.1	C7(Carbamidomethyl); C8(Carbamidomethyl)	0,0000	24	0,006374541	3	1807,79587	1,87	31,44	1

Supplementary References

1. Gibbs, D.J., Bacardit, J., Bachmair, A. & Holdsworth, M.J. The eukaryotic N-end rule pathway: conserved mechanisms and diverse functions. *Trends Cell Biol* 24, 603-611 (2014).
2. Tasaki, T., Sriram, S.M., Park, K.S. & Kwon, Y.T. The N-end rule pathway. *Annu Rev Biochem* 81, 261-89 (2012).
3. Varshavsky, A. The N-end rule pathway and regulation by proteolysis. *Protein Sci* 20, 1298-1345 (2011).
4. Garman, E.F. & Grime, G.W. Elemental analysis of proteins by microPIXE. *Prog Biophys Mol Biol* 89, 173-205 (2005).
5. Kallen, R.G. The mechanism of reactions involving Schiff base intermediates. Thiazolidine formation from L-cysteine and formaldehyde. *J Am Chem Soc* 93, 6236-48 (1971).
6. Giglione, C., Boularot, A. & Meinnel, T. Protein N-terminal methionine excision. *Cell Mol Life Sci* 61, 1455-74 (2004).
7. Ross, S., Giglione, C., Pierre, M., Espagne, C. & Meinnel, T. Functional and developmental impact of cytosolic protein N-terminal methionine excision in Arabidopsis. *Plant Physiol* 137, 623-37 (2005).
